# Window Technique for the Final Impression of a Maxillary Flabby Ridge: A Case Report of an Edentulous Patient

**DOI:** 10.7759/cureus.77508

**Published:** 2025-01-15

**Authors:** Syed Habib, Waleed S Alshehri, Saleh Alyousef

**Affiliations:** 1 Dentistry, King Saud University, Riyadh, SAU

**Keywords:** complete denture, edentulous patient, final impression, prosthodontics, window technique

## Abstract

The window technique is a highly useful clinical procedure for resolving several issues while taking a final impression of a patient with a mobile or displaceable anterior maxillary ridge, also referred to as a flabby ridge. This kind of ridge is particularly problematic due to the underlying tissues lacking strength and resilience, making it challenging to achieve a solid and comfortable denture fit. This typically exacerbates the problem, as conventional pressure during impression-making induces further displacement, which reduces the impression's precision. However, due to the utilization of a unique type of window in this impression technique, the window approach does not exert direct pressure on the flabby tissue. This innovative method of combining two types of impression materials, i.e., zinc oxide eugenol paste and impression plaster, with a window technique facilitates the fabrication of functional and well-fitting dentures by improving impression accuracy while reducing patient discomfort. The method outlined makes it easier for ordinary dentists to treat flabby ridge cases, allowing them to deliver patients with comprehensive care without the need for specialist referrals.

## Introduction

For patients who have lost all of their teeth, removable dentures remain an excellent choice, primarily because of current economic conditions. This is particularly detrimental to patients with low bone mass and structural defects [1,2]. By choosing a denture to replace the missing tissues, the complete denture seeks to provide comfort and a pleasing appearance. In order to improve stability, the denture is specifically made to be supported by the remaining alveolar ridge, which is covered with masticatory mucosa that is 1.5-2 mm thick [3]. Dentures require precise impressions of their bearing and limiting areas in order to perform well and function at their best. Wearing an improperly fitting complete denture can result in serious alterations of oral discourse and even inflict injury to the soft tissues [4]. Several injuries associated with using an improperly set removable prosthesis include denture stomatitis, disinserted fibromucosa, angular cheilitis, or "floating ridge," and fibrous hyperplastic lesion [5,6].

In dentistry, flabby ridges, also known as moveable or displaceable ridges, are a common clinical problem. This phenomenon, known as "combination syndrome," develops when an edentulous ridge conflicts with the native dentition or teeth [7]. The stability, support, and retention of dentures may be adversely affected by an excess of soft tissue in edentulous places. According to numerous studies, approximately 24% of edentate maxillae and 5% of edentate mandibles have flabby ridges [8]. Flabby ridges are easily dislocated under occlusal pressures due to inadequate support, which reduces denture retention and eliminates the peripheral seal [9]. The accuracy of the final imprint is compromised during the traditional/conventional final impression-forming process because the excessive and displaceable soft tissue is often compressed [10].

Numerous strategies have been offered for managing the flabby ridges. A hard denture-bearing area may be created by surgically excising the soft tissue that can be replaced. Patients may experience pain and discomfort from this approach, and their vestibular height may fall, reducing denture retention's effectiveness. In addition to being an expensive choice for patients, implant-retained prostheses, which rely on bone support instead of soft tissue, are not a viable treatment option due to the poor quality of the bone beneath the flabby ridges [11]. Other standard prosthodontic techniques, such as balancing occlusal loads and modified impression technique, are more commonly used, particularly for individuals with poor general health or financial circumstances [12].

The issue with flabby ridges is that they are susceptible to rebound and can cause the denture underneath to come loose because they were imprinted during the impression-taking procedure [13]. Therefore, to provide the best support while avoiding the displacement of flabby tissues, an impression technique that compresses the non-flabby tissues is necessary [14]. Notwithstanding these advantages, it is still unknown how well complete dentures made using the window impression approach will function clinically over the long run. To offer conclusive proof for its broad use, more clinical studies with longer follow-up periods are required. This case study describes the use of the window impression technique for prosthodontic rehabilitation of an edentulous patient with a maxillary anterior flabby ridge.

## Case presentation

**Figure 1:**
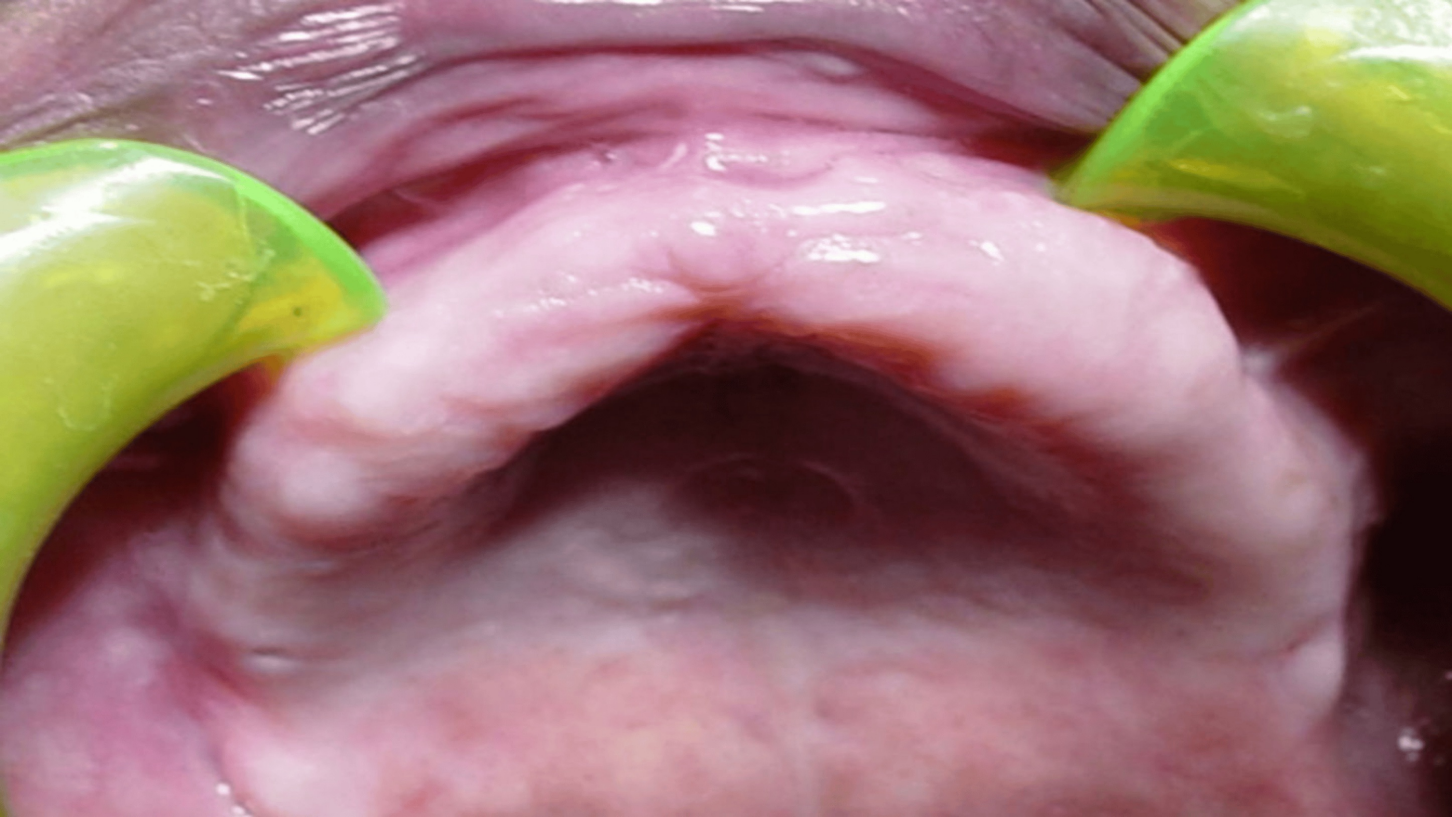
Pre-treatment intraoral picture of the maxillary edentulous arch with an anterior flabby ridge

A 62-year-old male, a controlled diabetic with no other significant medical conditions, presented in the prosthodontic clinic with a chief complaint of an ineffective old complete denture exhibiting poor retention. The extra-oral examination revealed no abnormalities. The intra-oral examination showed an edentulous maxillary arch with a displaceable anterior flabby ridge, in opposition to an anterior dentate mandibular arch (Figure 1). The patient was not ready for any surgery, including ridge augmentation or implant insertion, for the flabby ridge despite being informed of alternative treatment options. Finally, the patient was given the option for the fabrication of an upper maxillary complete denture fabricated using a special impression technique, i.e., the window technique, and the patient agreed to this treatment option. The patient was scheduled for new complete denture appointments and was instructed to discontinue using the old denture. Additionally, he was advised to massage the soft tissues with his fingers for a week to reduce any hyperplasia before commencing the clinical steps for the fabrication of a new denture.

Steps of the window technique impression

**Figure 2:**
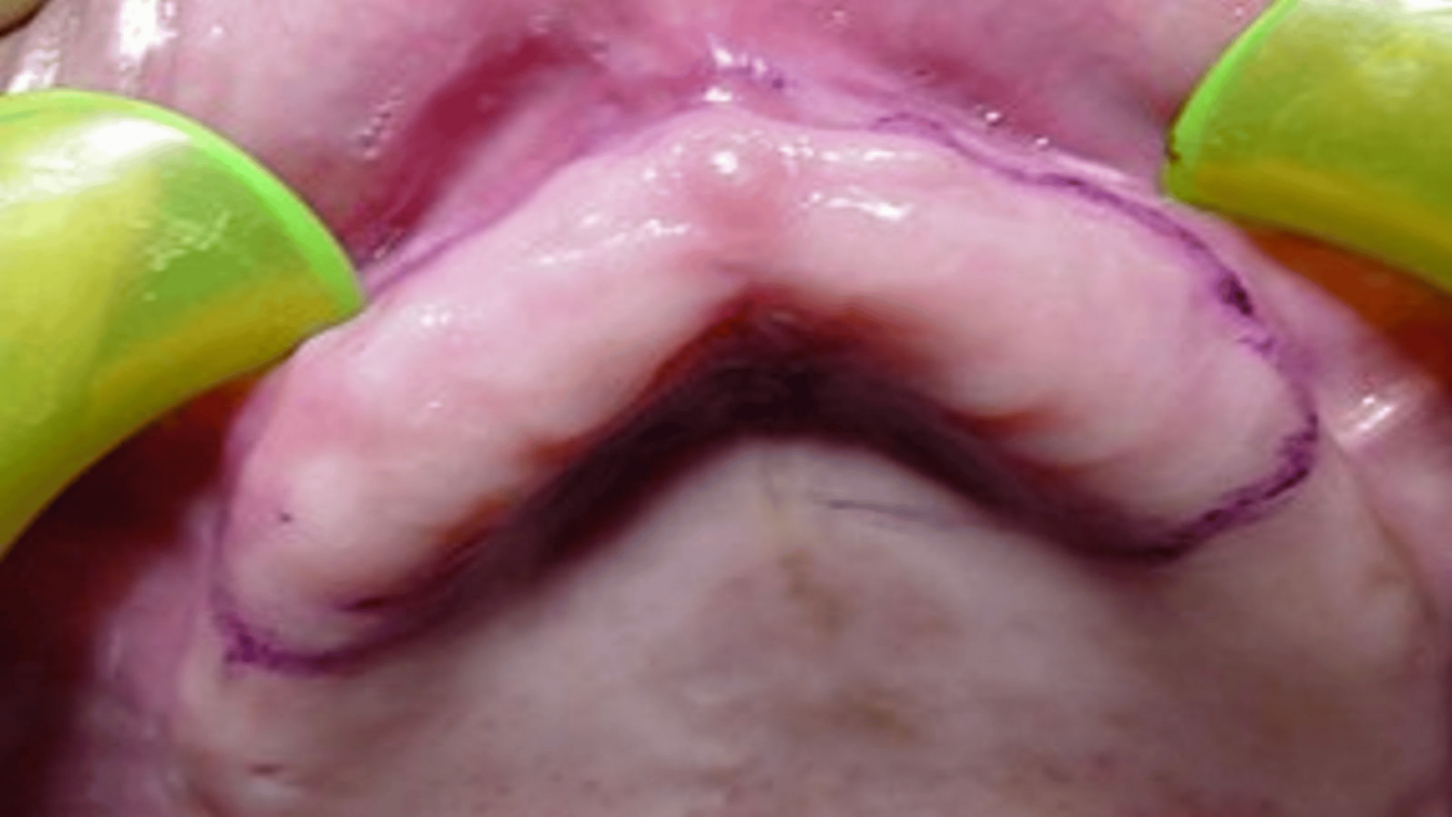
Flabby ridge area marked with an indelible pencil

A primary/initial impression was made with alginate (Zhermack dust-free thixotropic Tropicalgin, Zhermack SpA, Italy) material using edentulous stock trays. The flabby ridge area was marked with an indelible pencil intraorally (Figure 2). The alginate impression was reinserted in the patient's mouth, and the area marked with an indelible pencil was transferred to the impression.

**Figure 3:**
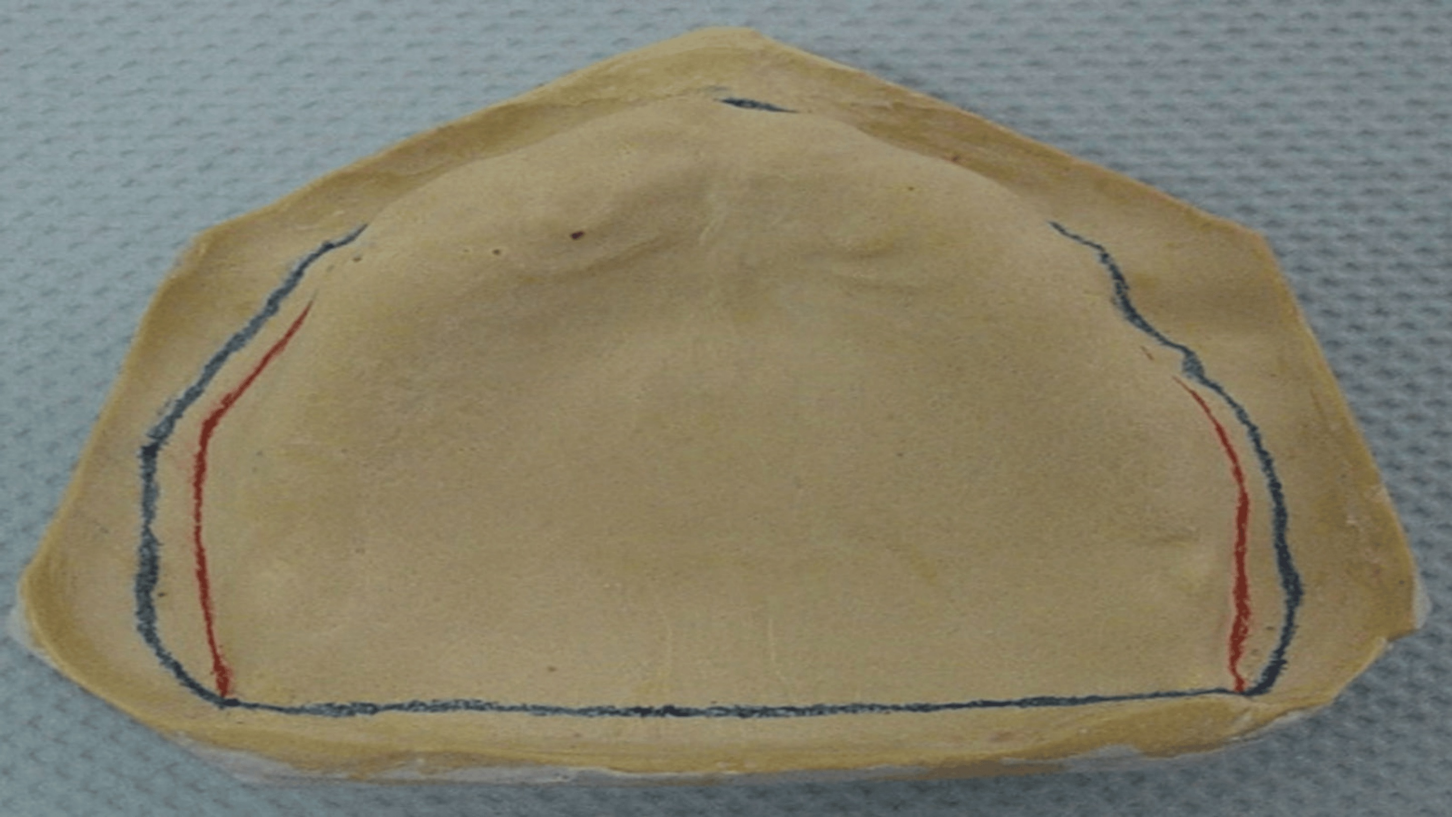
Maxillary primary cast

**Figure 4:**
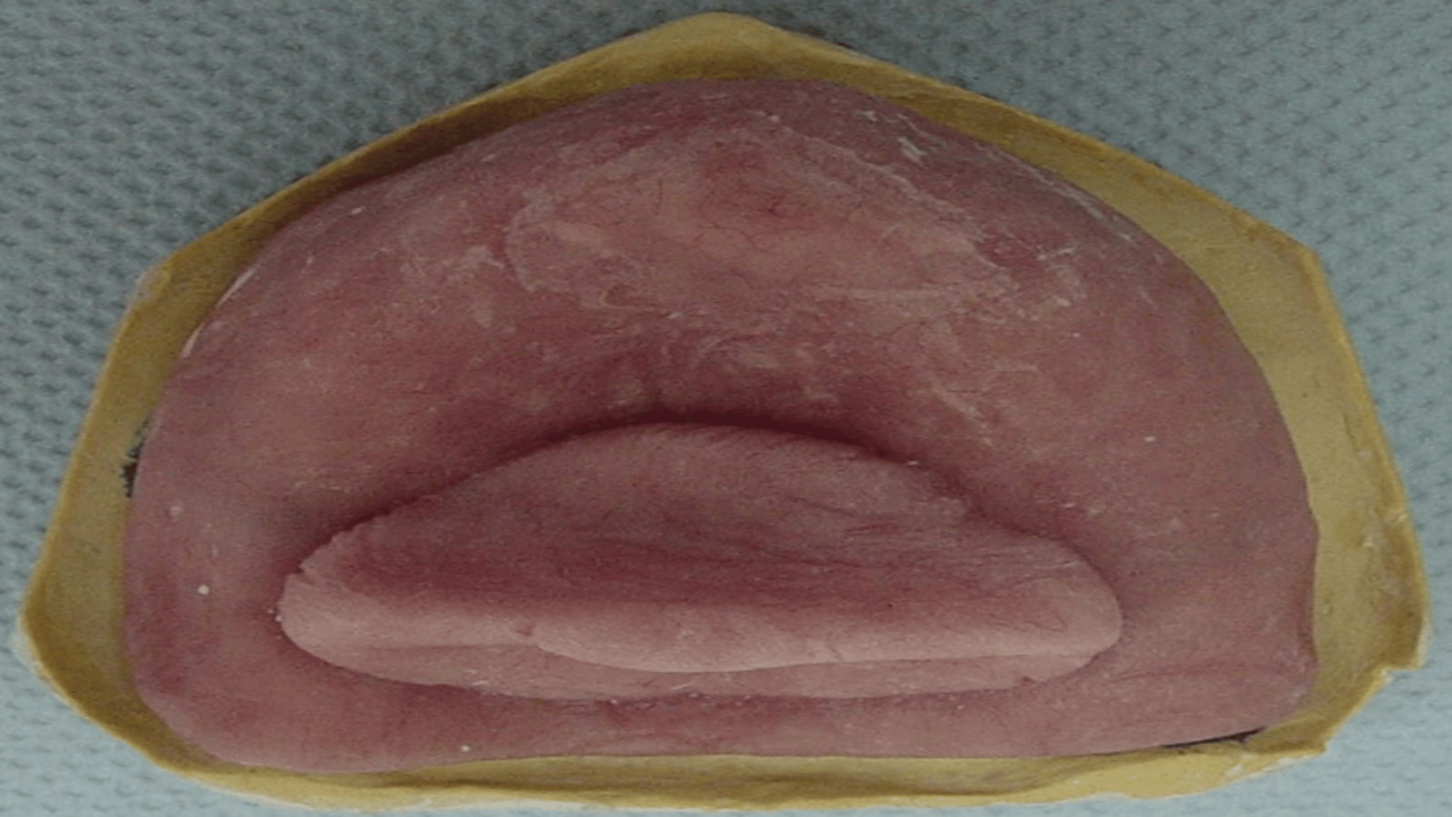
Upper custom tray with a handle in the mid-palate area

A maxillary cast was poured and fabricated (Figure 3) with dental stone (Dental Stone type III, Kulzer, Germany). A custom tray with a modified handle in the mid-palate area was fabricated with a 2 mm spacer, using a single sheet of modeling wax as a spacer and tissue stops (Figure 4).

**Figure 5:**
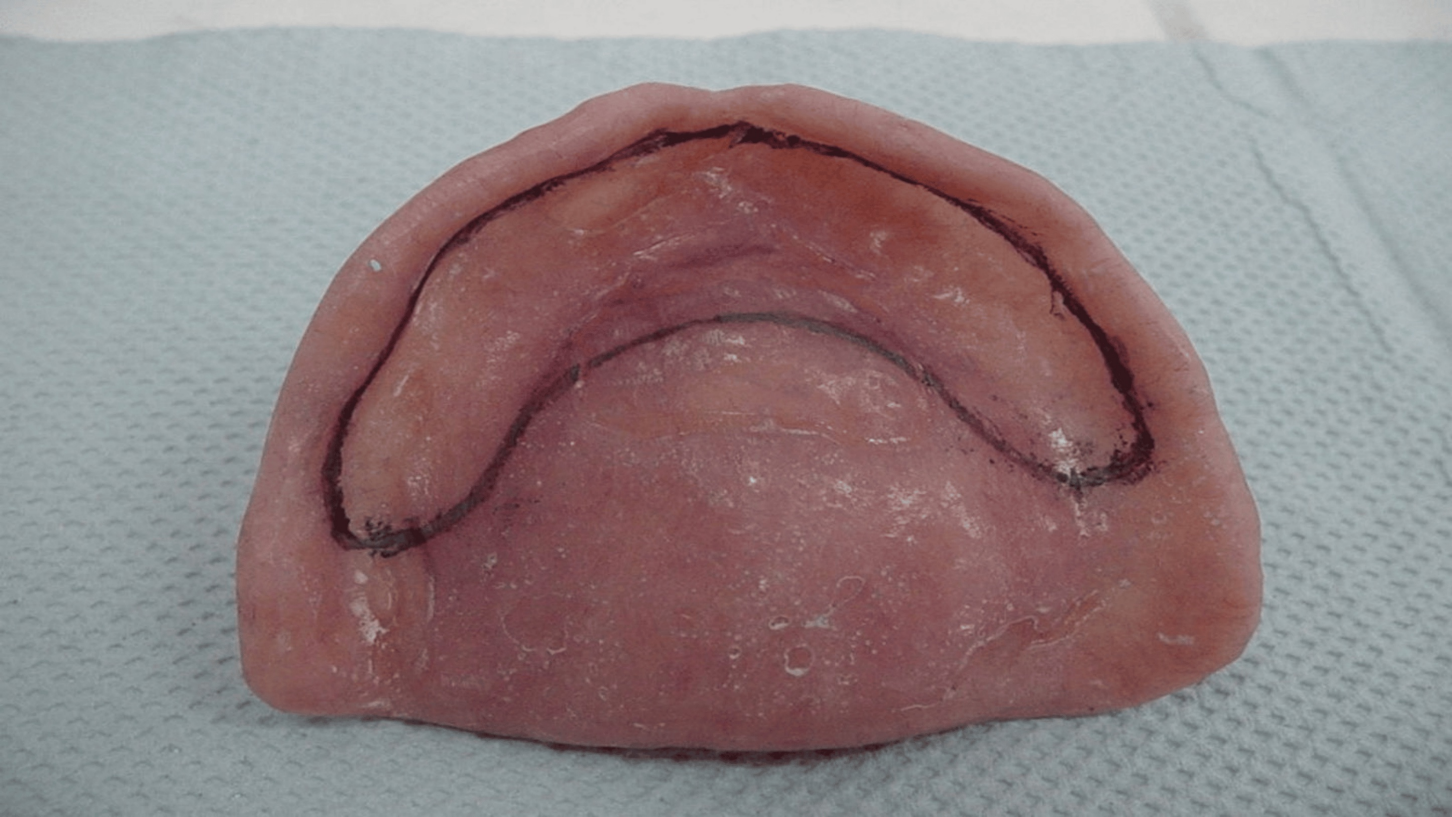
Outline of the flabby area transferred to the custom tray

**Figure 6:**
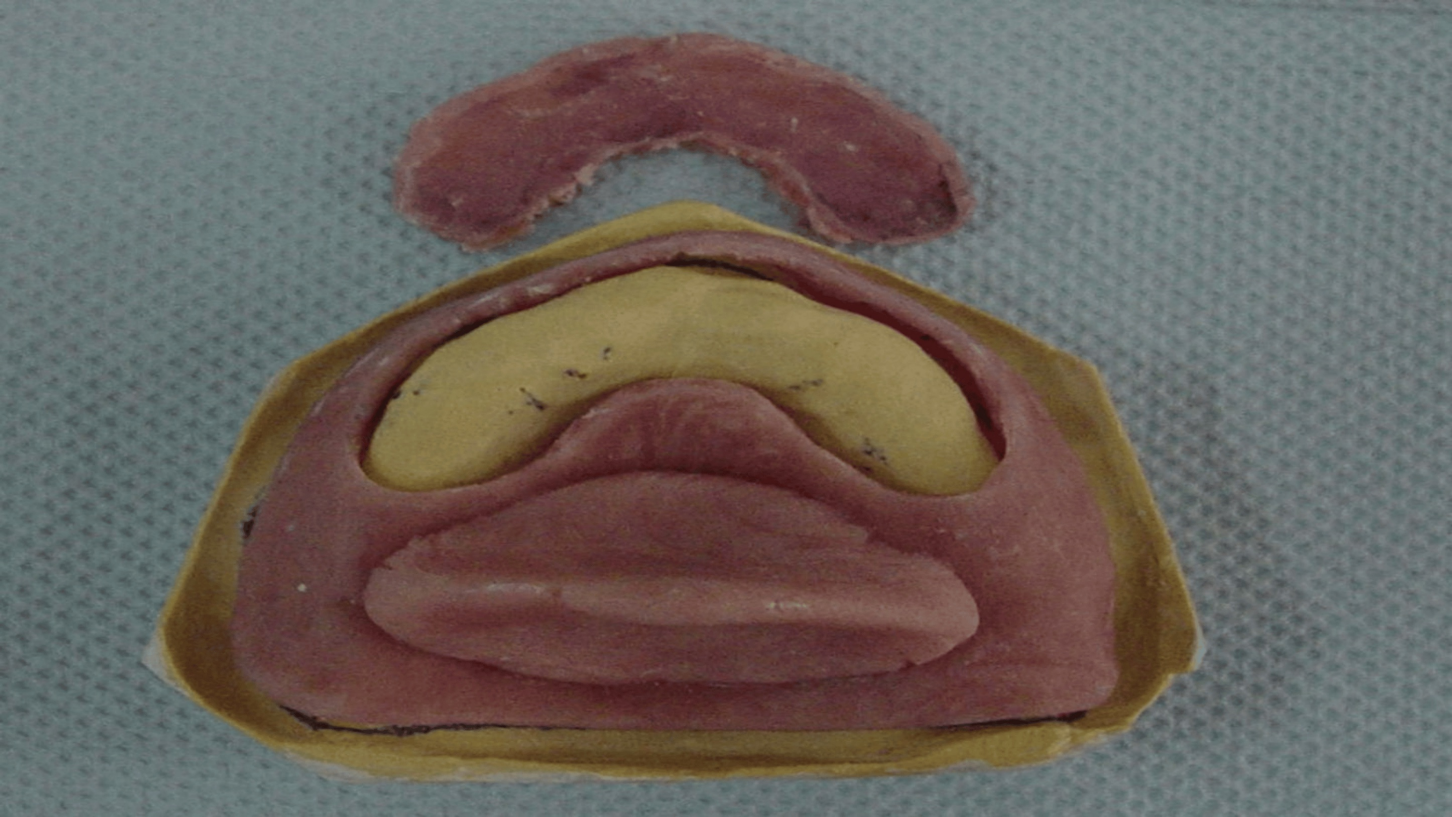
Window cut following the outline of the flabby area

**Figure 7:**
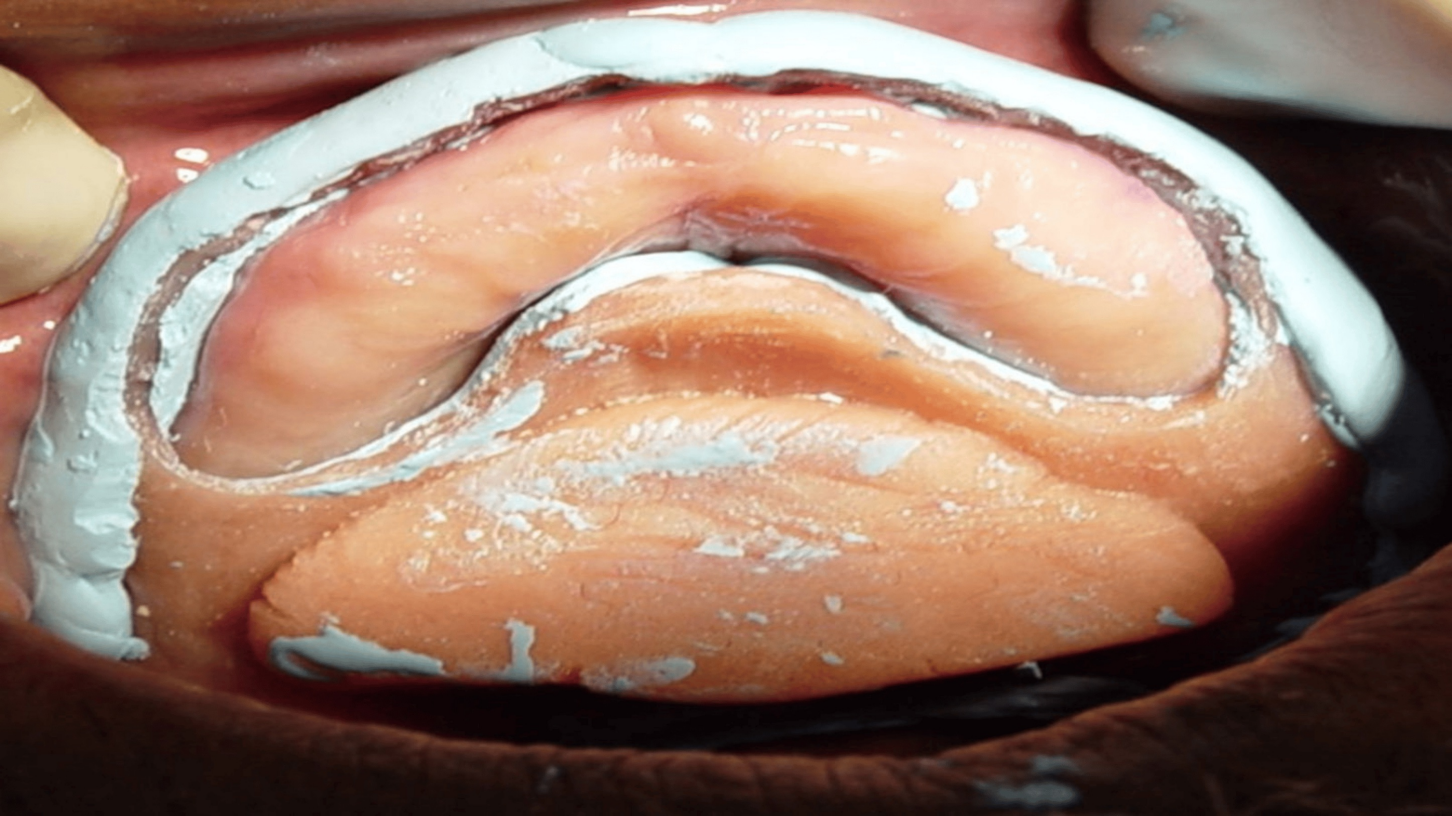
Tray with a window cut in the flabby ridge area, followed by an intraoral trial

Using the designated region surrounding the flabby ridge as a guide, a window was cut out of the tray (Figure 5). The window was cut to proceed with the border molding without compressing onto the flabby ridge tissue area, which was supposed to be covered with impression plaster later. A round-fissured bur was used to construct a window in the flabby tissue section of the customized tray (Figure 6). The tray was examined in the mouth to ensure the flabby area could be seen via the window (Figure 7).

**Figure 8:**
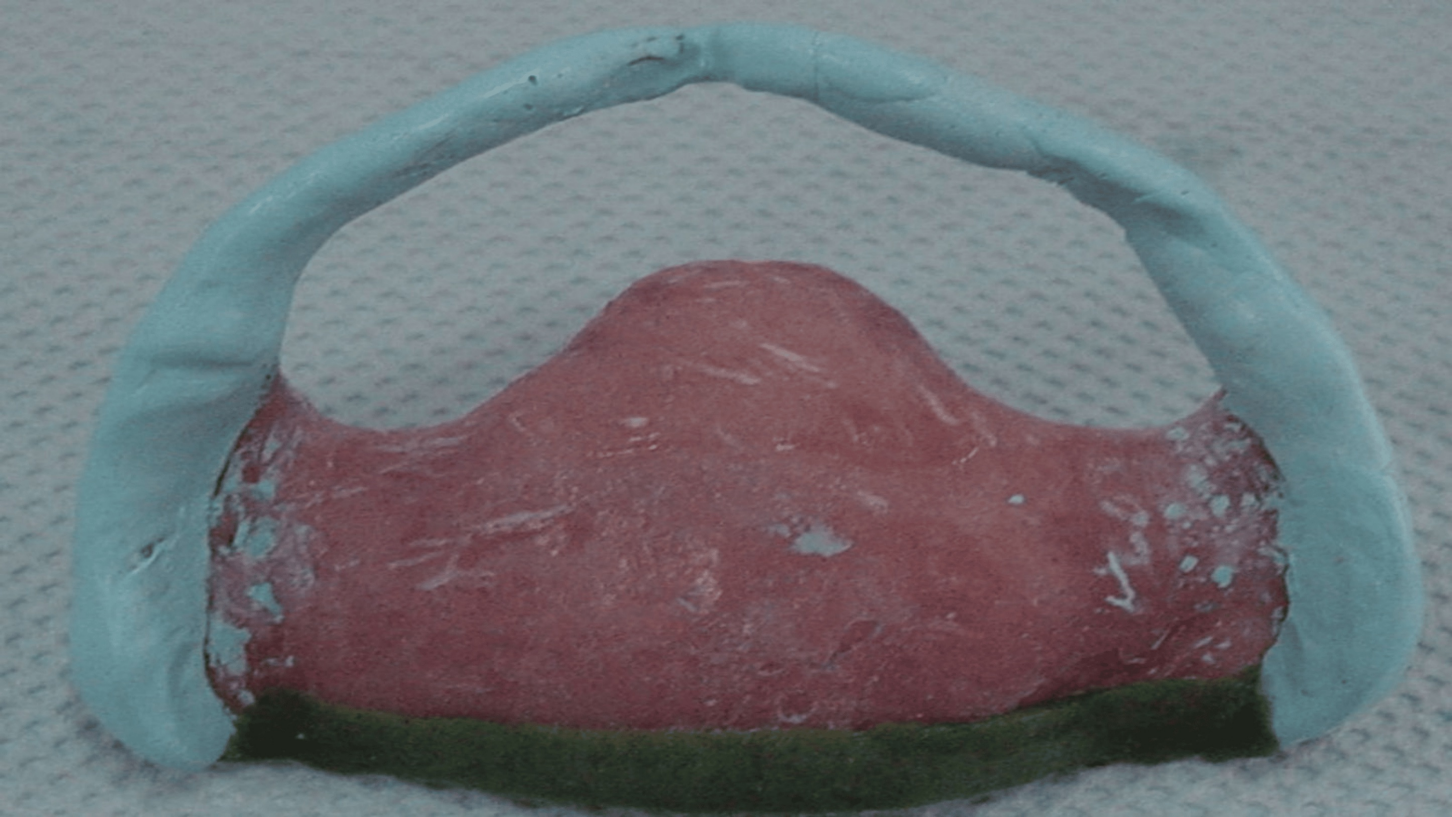
After border molding and washing impression of the peripheries

Using the selective pressure impression theory, the tray's edges were trimmed to be 2 mm below the sulcus. A low-fusing impression compound was used for border molding. Additionally, using zinc oxide eugenol paste (Kelly's ZOE impression paste, Water Pik, Inc., USA), a thin wash imprint of the peripheries was captured (Figure 8).

**Figure 9:**
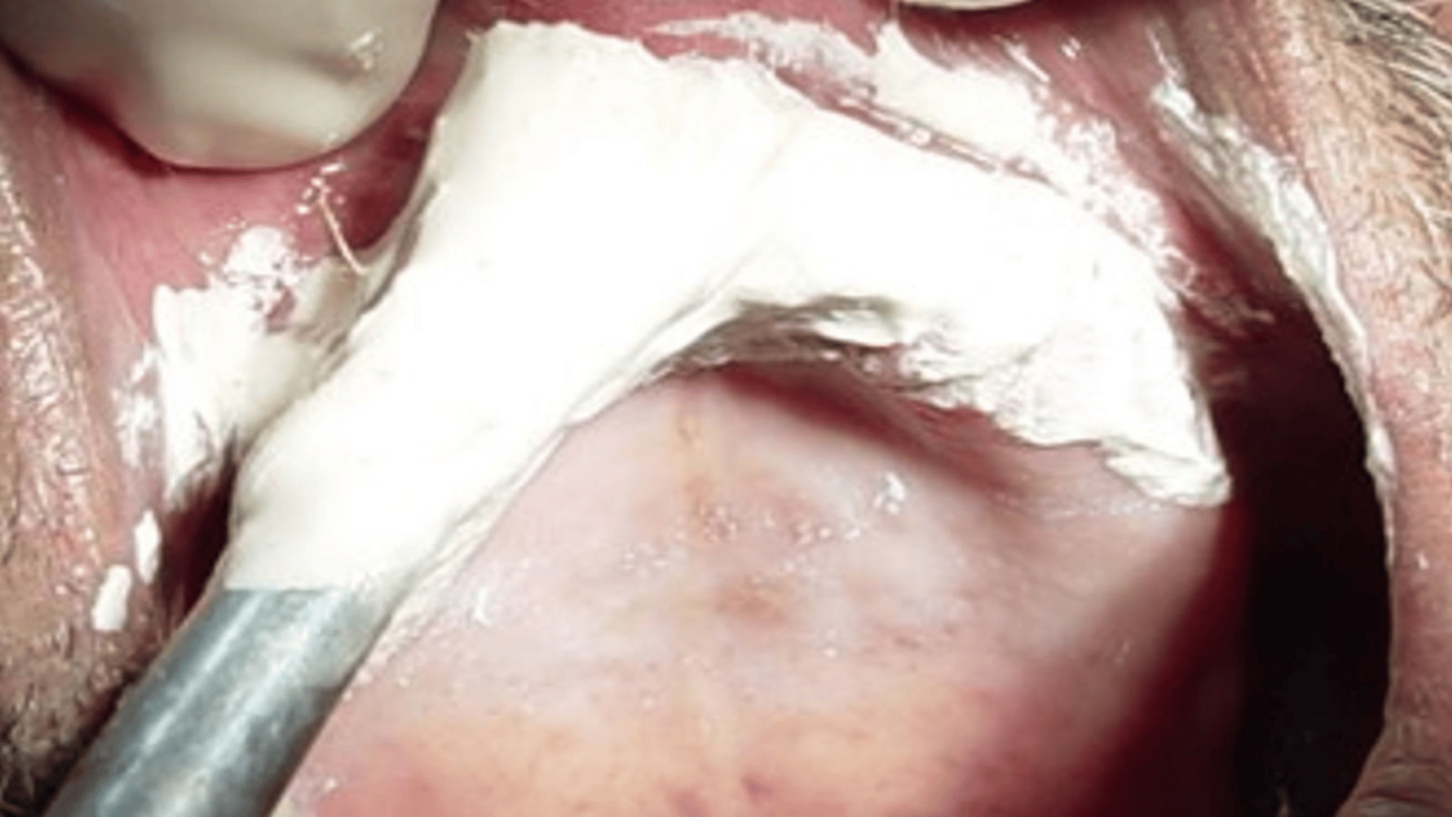
Painting of the impression plaster on the anterior flabby ridge area

**Figure 10:**
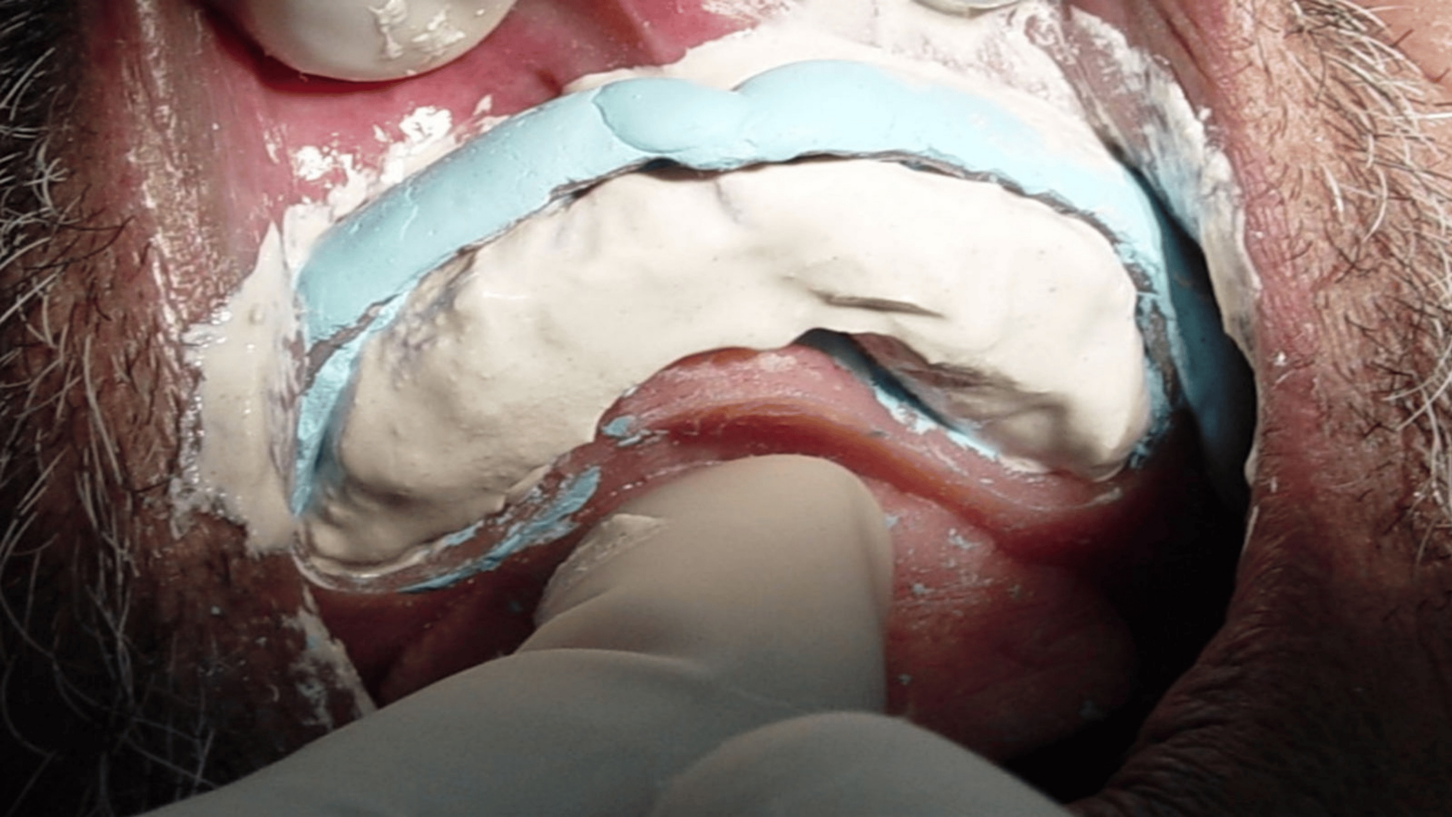
Tray insertion after the painting/application of the impression plaster

**Figure 11:**
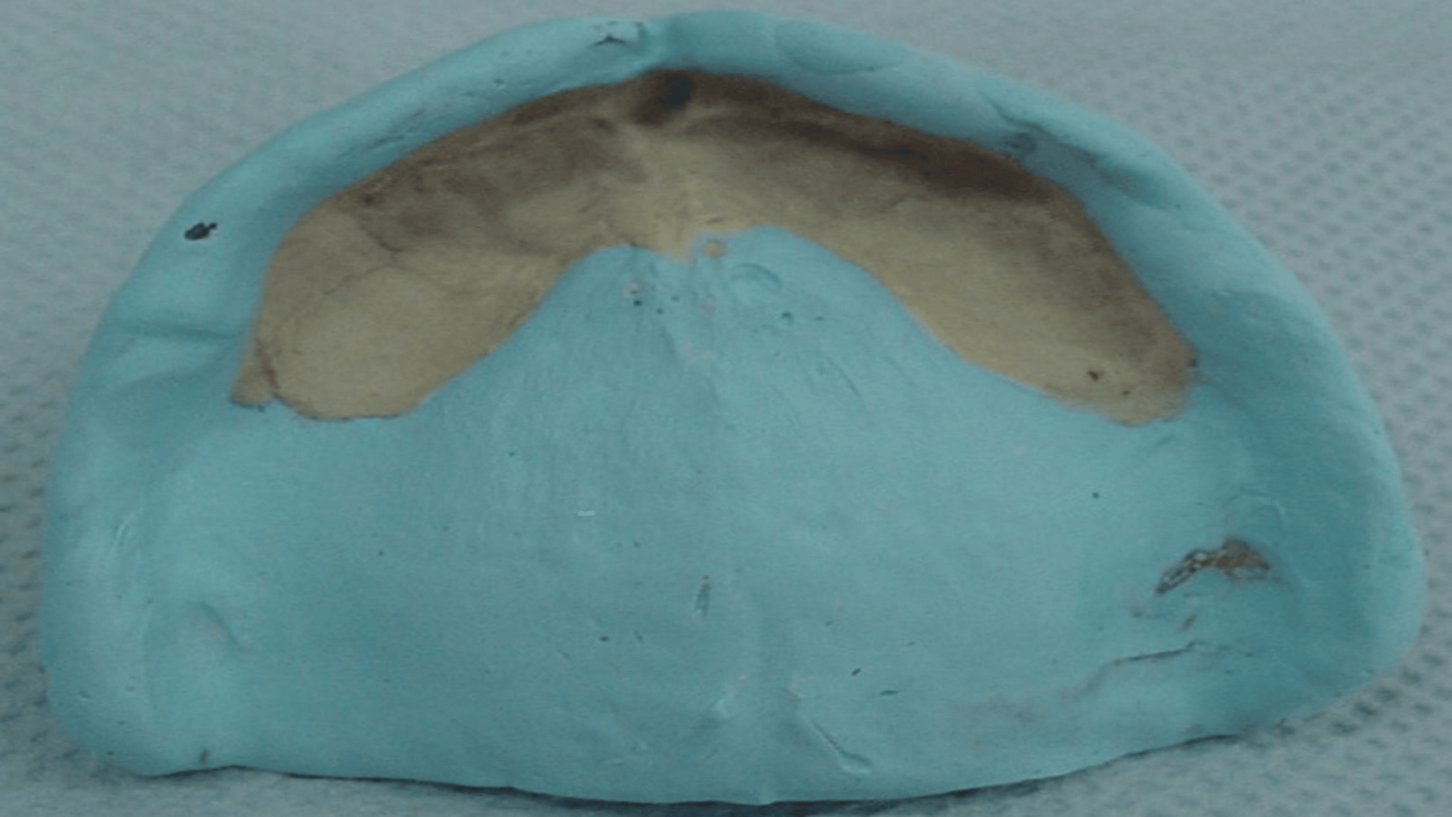
Final recorded impression with the window technique

A thin layer of impression plaster was painted/coated over the flabby ridge area with a soft camel hair brush (Figure 9). Then, the prepared impression tray, loaded with zinc oxide eugenol impression paste, was inserted over the painted impression plaster (Figure 10). The material was allowed to set completely, and then the impression was carefully removed from the patient's mouth (Figure 11).

After the final master cast was fabricated, the posterior palatal seal area was marked as a cupid bow shape and carved on the master cast. Subsequently, in accordance with standard protocol, the remaining steps of the entire denture manufacturing process were finished. After following post-insertion guidelines, the patient received his completed dentures. The upper complete denture's fit and retention pleased the patient.

## Discussion

When opposed to lower natural dentition, the therapeutic problems of restoring maxillary edentulous patients with anterior flabby ridges require careful planning. In order to guarantee successful outcomes and the long-term stability of the underlying tissues, dentists must choose the best course of treatment. Edentulous patients may benefit from an upper complete denture fabricated with a conventional impression technique. Those with anterior flabby ridges, as opposed to natural dentition, can benefit the most [13-15]. However, this is not recommended if the underlying mucosal tissues are abused or if there is an *Epulis fissuratum* or fibrous hyperplasia [7]. The benefits of the window technique as an alternative to traditional impression procedures are demonstrated by the successful placement of an anterior flabby ridge in an edentulous maxillary arch in an elderly male patient, as demonstrated in this case report. The patient's age, their wish to forego surgery, ridge augmentation, and implant treatment, as well as the restrictions of the traditional impression approach in this type of flabby ridge instance, all played a role in the decision to employ the window technique. The treatment aimed to create a denture with good retention, support, and stability and a denture to withstand the compressive masticatory forces of the opposing natural dentition.

Due to the fibrous soft tissue's elastic recoil during functioning, conventional impression procedures compress flabby ridges, resulting in instability, loss of denture retention, and dislodging [15]. Although special treatment techniques are frequently required for flabby ridges, treatment planning for these conditions necessitates a complete revision of the treatment strategy. According to a careful review of the literature, impression techniques and materials account for the majority of the changes made to the conservative management of flabby ridges. In addition to the continuous debate between mucostatic and selective impression techniques, the literature also addresses the use of modified or alternative procedures and the use of other materials [16]. Several techniques have been documented, including spacers or perforations [17], sectional trays, and split trays [18]. Nevertheless, there is no proof that one method is better than another for producing a stable and retentive denture on flabby ridges [19].

The window technique for taking an impression of the anterior maxillary flabby ridge is explained in this article. The method Watson describes is somewhat similar to the one described here [19]. Normal tissues are subjected to a mucocompressive/selective pressure impression technique, and the tissue is captured in its static state by the impression plaster covering the window. Liddelow proposed zinc oxide, eugenol, and impression plaster in 1964 [20]. Choosing a window is likely the best way to ensure that the flabby tissue is not under any pressure. Recent studies on various tray designs have shown that the tray with a window causes the fewest tissue alterations [13]. Using impression plaster during the imprinting process may cause the tissues to be compressed or moved. The flabby tissue can fully return while the plaster is setting, as the tray design includes a window to prevent the plaster from being restricted to the tray [6,9]. Additionally, the therapist can observe how the impression material is adjusting to the flabby tissue due to the open tray. It is crucial that the flabby area be recorded while at rest, without any compression or displacement. Another benefit of the window is that, even under pressure, the flabby tissue can return to its resting position. Therefore, a window and impression materials such as impression plaster are crucial for documenting the resting condition of a flabby ridge. Moreover, this modified impression method decreases chairside time and the requirement for extra clinical steps/appointments. This easy, affordable, and simple method can reduce the amount of movement of the flabby tissue during the impression procedure.

More research with longer follow-up periods is needed to definitively ascertain the efficacy and longevity of dentures made using the impression process described in this case report. This case study adds to the growing corpus of evidence demonstrating that special impression techniques, such as the window technique, can be employed to obtain a final impression of the flabby maxillary ridge while fabricating complete dentures. The authors recommend the practical use of this window technique.

## Conclusions

This case study shows how the window technique can be used to successfully record a patient's final impression when they have an edentulous maxillary anterior flabby ridge. With this minimally invasive method, the impression can be taken with little movement of the flabby ridge, reducing cost and yielding good retention and masticatory performance results. The present case contributes to the mounting evidence supporting unique impression techniques for edentulous patients, even though further long-term studies are necessary. It must be emphasized that the efficacy of this therapy modality depends on a careful case selection procedure and careful impression technique implementation.
